# Development and Validation of a Nomogram for Predicting the Mortality after Penetrating Traumatic Brain Injury

**DOI:** 10.29252/beat-070402

**Published:** 2019-10

**Authors:** Thara Tunthanathip, Suphak Udomwitthayaphiban

**Affiliations:** 1 *Division of Neurological Surgery, Department of Surgery, Faculty of Medicine, Songklanagarind Hospital, Prince of Songkla University, Songkla, Thailand*; 2 *Division of Neurological Surgery, Department of Surgery, Faculty of Medicine, Songklanagarind Hospital, Prince of Songkla University, Songkla, Thailand *

**Keywords:** Penetrating brain injury, Traumatic brain injury (TBI), Nomogram

## Abstract

**Objective:**

To determine the factors associated with mortality in penetrating brain injury (PTBI) and proposed the nomogram predicting the risk of death.

**Methods:**

A retrospective cohort study was conducted on all patients who had sustained PTBI between 2009 and 2018. Collected data included clinical characteristics, neuroimaging findings, treatment, and outcomes. Prognostic factors analysis was conducted using a forest plot. Therefore, the nomogram was developed and validated. For the propose of evaluation, the nomogram’s sensitivity, specificity, positive predictive value (PPV), negative predictive value (NPV), Receiver Operating Characteristic (ROC) curve and the area under the receiver operating characteristic (AUC) were determined for validating the optimal cut-off point of the total scores.

**Results:**

During the study period, 62 individuals enrolled. In the univariate analysis, factors associated with the morality were normal pupils’ reactivity to light (OR 0.04, *p* < 0.001), hypotension (OR 9.91, *p*<0.001), hypoxia (OR 10.2, *p*=0.04), bihemispheric injuries (OR 19.0, *p*=0.001), multilobar injuries (OR 21.5, p< 0.001), subarachnoid hemorrhage (OR 6.9, *p*= 0.02), intraventricular hemorrhage (OR 26.6, *p*= 0.006), basal cistern effacement (OR 28.8, , *p*<0.001), midline shift >5 mm (OR 0.19, *p*<0.001) were significantly associated with death. In multivariable analysis, hypotension (OR 8.82, *p*=0.03), normal pupils’ reactivity to light (OR 0.07, *p* =0.01), midline shift >5 mm (OR 18.23, *p*<0.007) were significantly associated with death. The nomogram’s sensitivity, specificity, PPV, NPV, and AUC for predicting mortality (total score ≥ 100) were 80%, 92.6%, 72.7%, 95.0%, and, 0.86 respectively.

**Conclusions:**

PTBI is the fatal injury depend on both clinical and neuroimaging parameters. The nomogram is the alternative method providing prognostic parameters toward implication for clinical decision making.

## Introduction

Penetrating traumatic brain injury (PTBI) is much less common than blunt traumatic brain injury [1]. Larkin *et al*. reported a prevalence of PTBI in 8.8% of TBI cases but this injury is significantly more severity and poor outcome [2]. The degree of injury of missile/flak is produced by both low-velocity and high-velocity projectiles according to the mass–energy equivalence ($E=mc^{2}$) [3,4]. Gunshot injuries are the most common cause PTBI and bring high mortality. The mortality rates from gunshot wounds range between 21% and 88%. [5-9]. 

Prognostic factors, which included clinical characteristics and neuroimaging features, have been reported in the literature. Bandt *et al*. proposed the St. Louis Scale for Pediatric Gunshot Wounds to the Head in 2012 [10] while Muehlschlegel *et al*. proposed the SPIN score in 2016. Similarly, these predictive scores based on physical examination, imaging findings, and laboratory [11]. Additionally, neuroimaging features have been mentioned for predicting mortality and decision-making treatment in an emergency situation [9-11].

Currently, the nomogram has been used to predict clinical outcome in various diseases such as oncology [12,13] and other diseases [14-16]. The model is a mathematical equation that joins the predictors and the outcome of interest with two-dimension graphic scale. The objectives of the current study were to identify various predictors associated with death in PTBI patients and to propose the nomogram to predict mortality. 

## Materials and Methods 

*Study designs and study population*

The authors conducted a retrospective review of the database of our trauma registry. We enrolled consecutive PTBI patients who were treated at the university trauma centers from 2009 through 2018. Several clinical, laboratory, treatment and radiological factors were collected for analysis. Additionally, patients were divided into three groups according to their initial Glasgow Coma Scale (GCS) score: mild TBI (GCS score 13–15), moderate TBI (GCS score 9–12), and severe TBI (GCS score 3–8).

On the basis of neuroimaging, the entry trajectory, intracranial injuries, pressure effect, and other characteristics were reviewed by two neurosurgeons. Bihemispheric injuries were defined as injuries in which the missile/flak track crossed the midsagittal plane, causing injury to both cerebral hemispheres. Multilobular injuries were defined as CT scan evidence of damage to more than one lobe of the brain. In the laboratory, hypoxia was defined as oxygen saturation < 92% or partial pressure of oxygen < 80 mm Hg and anemia was defined as a hemoglobin level below 9 g/dl at admission. According to Wu *et al*. and clinical practice at our institute, coagulopathy was defined as thrombocytopenia (platelet count < 100,000/µl) or elevated international normalized ratio > 1.2 or prolonged activated partial thromboplastin time >40 seconds at admission [17,18]. At the time of hospital discharge, the morality of the patients was considered as the primary outcome of the study. The study was performed with the approval of the Ethics Committee of the Faculty of Medicine, Songklanagarind Hospital, Prince of Songkla University (REC.62-012-10-1).

*Nomogram development and validation*

Using binary logistic regression analysis, the prediction model was constructed from the significant parameters that affect the mortality. A nomogram, which based on the binary logistic regression model, was developed using the significant parameters (*p* < 0.05) by Zhang *et al*. method [19]. The bootstrap method with 1000 replicates was used for the internal validity of the model. The validate function in the rms package was used to analyze the bias-corrected c-index that evaluated the predictive discrimination of the model. The c-index is the probability of concordance between predicted probability and response. For the practical insight, the nomogram was evaluated the optimal cut-off point for predictive the binary classifiers (death or living) instead of the death-probability prediction. Using the self-consistency validation, the scale’s sensitivity, specificity, positive predictive value (PPV), negative predictive value (NPV), accuracy was determined for death in various cut- off point of the total scores. Moreover, the Receiver Operating Characteristic (ROC) curve and the area under the receiver operating characteristic (AUC) were plotted. Additionally, AUCs were determined that values ≥0.9 are “excellent,” ≥0.80 “good,” ≥0.70 “fair,” and <0.70 “poor” [20,21].

*Statistical analysis*

The mean, with standard deviation, was calculated from descriptive purposes. The binary logistic regression analysis was used to identify the predictors of death both univariate and multivariable analysis. In multivariable analysis, the forward method was used to check whether predictors deserved to be included in the model. The statistical analysis was performed using the R version 3.4.0 software (R Foundation, Vienna, Austria). In details, the ‘ggplot2’package was used for building the forest plot of odds ratio with 95% confident interval (95%CI) [22]. The ‘rms’ package was used for creating nomogram [23]. Moreover, ROC and AUC were created by ‘PlotROC’ package [24].

## Results

*Clinical characteristics*

The baseline characteristics of the study population are presented in Table 1. The study involved 62 individuals enrolled. More than half of them were gunshot injury at the head, whereas penetrating injury from flak of explosion found more than one-third of them. Additionally, PTBI from suicidal attempt was 6.5% of cases while injury from secondary blast effect was 38.7%. The mean age of the study population was 37.7 (SD 15.8) years. The percentage of the male was 91.9% of the study population. Concerning severity, 40.3% of the cases were severe TBI, while 50.0% had mild TBI. The secondary brain insults, particularly hypotension and hypoxia, were observed at 35.5% and 8.1%, respectively. 14.5% of the PTBI patients, who developed persistent instability of their vital signs, did not acquire the CT of the brain. In neuroimaging parameters, two-thirds of cases had the coronal plane of penetrating trajectory while the frontal region was the most common entry site of bullets/flakes. Depressed skull fracture was the most common intracranial pathology observed in neuroimaging. Multilobar injuries, bihemispheric injuries, and perforating injuries were 20.7%, 16.9%, and 1.6%, respectively. Therefore, almost two-thirds of cases underwent to operations and mortality rate of the present study at hospital discharge was 32.3% of cases. 

*Factors associated with mortality*

As the results, the univariate analysis revealed that severe TBI (Odds ratio [OR] 80.7, *p*<0.001;), normal pupils’ reactivity to light (OR 0.04, *p*<0.001), hypotension (OR 9.91, *p*<0.001), Hypoxia (OR 10.2, *p*=0.04), bihemispheric injuries (OR 19.0, *p*=0.001), multilobar injuries (OR 21.5, *p*< 0.001), subarachnoid hemorrhage [SAH] (OR 6.9, *p*= 0.02), intraventricular hemorrhage [IVH] (OR 26.6, *p*= 0.006), basal cistern effacement (OR 28.8, *p*<0.001), midline shift >5 mm (OR 19.0, *p*<0.001) were significantly associated with death as shown in Table 2. In multivariable analysis, hypotension (OR 8.82, *p*=0.03), normal pupils’ reactivity to light (OR 0.07, *p* =0.01), midline shift >5 mm (OR 18.23, *p*<0.007) were significantly associated with death by backward stepwise method as shown in Table 3. 

**Fig. 1:**
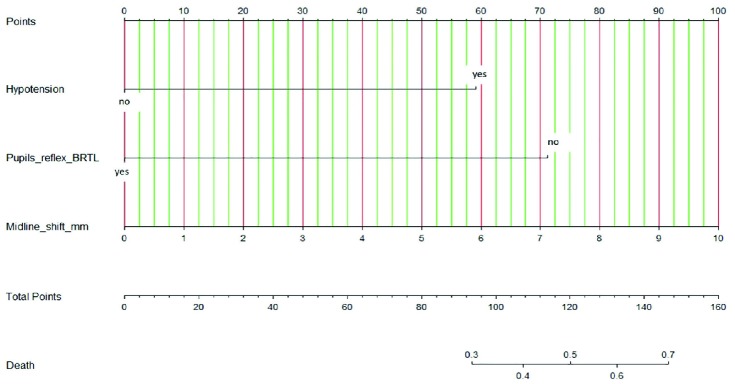
Nomogram predicting the mortality in penetrating traumatic brain injury. To use the nomogram, draw a straight line upward from the patient's characteristics such as SHI, pupils reflex, SAH, IVH to the upper points scale, the sums of the scores of all variables. Then, draw another straight line down from the scale of the total points through the risk of death. This is the probability of the presence of death in an individual. Abbreviation: BRTL; Both pupils react to light, mm; millimeter

**Fig. 2:**
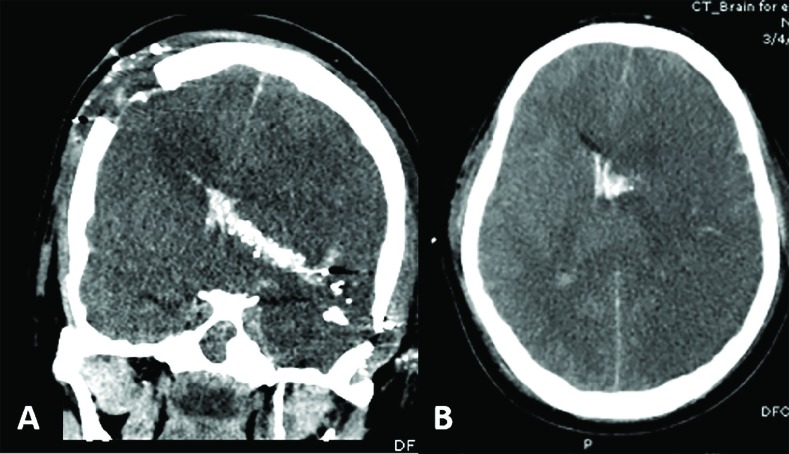
Perforating injury. (A) Coronal plane CT of the brain shows bullets pass at the right parietal area and cross through multilobar involvement. Finally, the bullets exit through at left temporal area. (B). Axial CT of the brain showed diffuse brain swelling, intraventricular hemorrhage, and subarachnoid hemorrhage

**Fig. 3:**
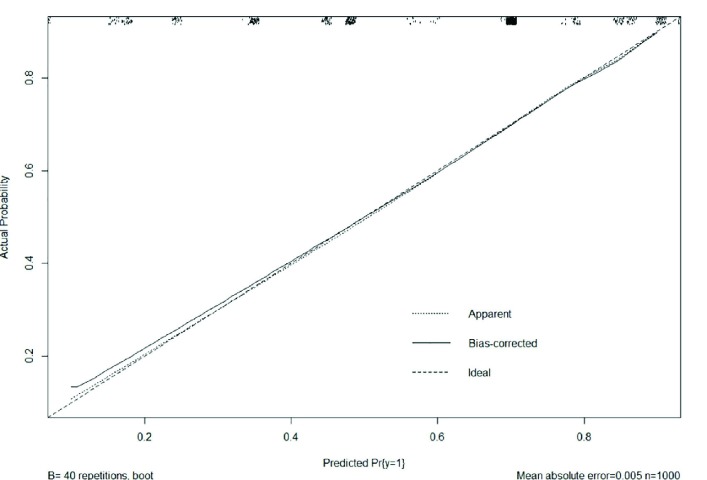
Bootstrapped calibration plot, which proves that concordance between the predicted probability and response is satisfactory

**Fig. 4:**
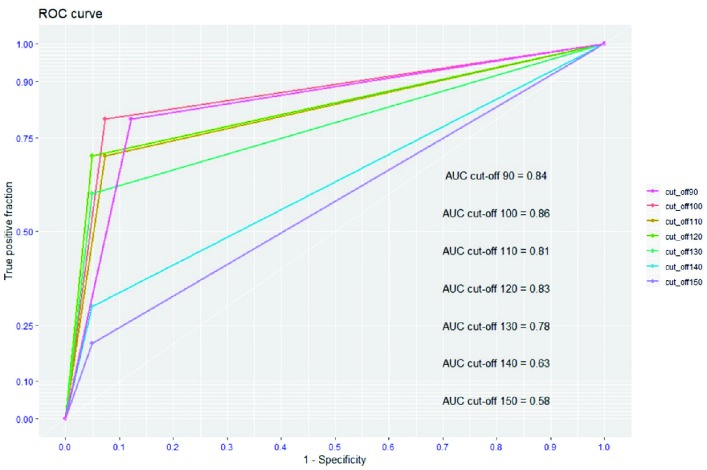
The Receiver Operating Characteristic (ROC) curve and the area under the receiver operating characteristic (AUC) of each cut-point of the nomogram

**Table 1:** Clinical characteristics

**Factor**	**N (%)**
**Gender**	
Male	57 (91.9)
Female	5 (8.1)
**Mean of age** (SD)-year	37.7 (15.8)
**Military personnel**	15 (24.2)
**Mechanism **	
Gunshot injury	35 (56.5)
Secondary blast injury	24 (38.7)
Sharp object injury	3 (4.8)
**Suicide **	4 (6.5)
**Glasgow Coma Scale score**	
13-15	31 (50)
9-12	6 (9.7)
3-8	25 (40.3)
**Pupillary size and light reflex **	
Fixed and dilated both eyes	15 (24.2)
Unequal	5 (8.1)
Normal and reflex both eyes	42 (67.7)
**Hypotension (Blood pressure <90/60) **	22 (35.5)
**Anemia**	15 (24.2)
**Coagulopathy**	13 (21.0)
**Hypoxia**	5 (8.1)
**Acidosis **	3 (5.3)
**Mean of neutrophil/lymphocyte ratio** (SD)	8.3 (8.1)
**CT scan of the brain**	
No (Unstable)	9 (14.5)
Yes	53 (85.5)
**Plane of track **	
Coronal	24 (47.1)
Sagittal	27 (52.9)
**Entry site** (N=51)	
Frontal	19 (37.3)
Temporal	9 (17.6)
Parietal	9 (17.6)
Occipital	8 (15.7)
Maxillary	4 (7.8)
Orbital	2 (3.9)
**Pathology **(N=53)	
Perforating injury	1 (1.6)
Bihemispheric injury	9 (16.9)
Multilobar injury	11 (20.7)
Linear skull fracture	6 (11.3)
Depressed skull fracture	32 (60.4)
Basilar skull fracture	10 (18.9)
Epidural hematoma	8 (12.9)
Subdural hematoma	22 (41.5)
Contusion	24 (45.3)
Brainstem	1 (1.6)
Subarachnoid hemorrhage	23 (43.4)
Intraventricular hemorrhage	5 (9.43)
Cistern effacement	13 (24.5)
Diffuse brain edema	9 (17.0)
Mean of midline shift (SD)-mm	1.6 (2.9)
**Surgery**	
No	25 (40.3)
Decompressive craniectomy with debridement	12 (19.4)
Craniotomy with debridement	24 (38.7)
Mastoid approach for foreign body removal	1 (1.6)
**Discharge mortality **	20 (32.3)

**Table 2:** Univariate analysis for the mortality in patients with penetrating brain injury

**Factor **	**Odds ratio (95%CI)**	**p-value**
**Glasgow Coma Scale score**		
9-15	Ref	
3-8	80.7 (9.37-695.53)	<0.001
**Pupil reactivity**		
Non-BE	Ref	
BE	0.04 (0.01-0.17)	<0.001
**Laboratory**		
Anemia	3.20 (0.91-11.24)	0.06
Hypotension	9.91 (2.90-33.85)	<0.001
Hypoxia	10.2 (1.06-98.83)	0.04
Coagulopathy	7.7 (2.00-30.14)	0.003
**Plane of track**		
Coronal	Ref	
Sagittal	0.86 (0.21-3.44)	0.83
**Neuroimaging findings**		
Bihemispheric injuries^a^	19.0 (3.37-106.84)	0.001
Multilobar injuries^a^	21.5 (3.93-118.26)	<0.001
Subarachnoid hemorrhage^a^	6.9 (1.29-37.0)	0.02
Intraventricular hemorrhage ^a^	26.6 (2.53-280.5)	0.006
Subdural hemorrhage^a^	2.42 (0.65-9.01)	0.18
Contusion ^a^	0.82 (0.22-3.03)	0.77
Basal cistern effacement ^a^	28.8 (4.71-175.97)	<0.001
**Midline shift**		
< 5 mm	Ref	
> 5 mm	19.0 (3.3-106.84)	<0.001

^a^ Data show only “yes group” while reference groups (no group) are hidden.

**Table 3:** Multivariate analysis

**Factor **	**Odds ratio (95%CI)**	***p*** ** value **
**Hypotension**		
No	Ref	
Yes	8.82 (1.11-69.85)	0.03
**Pupil reactivity **		
Non-BE	Ref	
BE	0.07 (0.01-0.62)	0.01
**Midline shift**		
< 5 mm	Ref	
> 5 mm	18.23 (2.22-149.52)	0.007

**Table 4:** Optimizing cut-points for prediction of mortality

**Cut-off point **	**Sensitivity**	**Specificity**	**PPV** ^a^	**NPV** ^b^	**Accuracy**	**AUC** ^d^
90	80.0	87.8	61.5	94.7	86.2	0.84
**100**	**80.0**	**92.6**	**72.7**	**95.0**	**90.2**	**0.86**
110	70.0	92.6	70.0	92.6	88.2	0.81
120	70.0	95.1	77.7	92.8	90.2	0.83
130	60.0	95.1	75.0	90.7	88.2	0.78
140	70.0	95.2	77.7	92.8	90.2	0.62
150	80.0	95.1	80.0	95.1	92.1	0.57

^a^PPV: positive predictive value, ^b^NPV: negative predictive value, ^c^ROC: Receiver Operating Characteristic curve, ^d^AUC: area under the receiver operating characteristic

*Nomogram development and validation*

As show in Figure 1, the use of the nomogram is simple. For example, a 45-year-old male suffered from gunshot wound at the head. At emergency department, his clinical characteristics were GCS score 3, bilateral fixed and dilated pupils (71 points), initial blood pressure was 120/70 mmHg (0 points). After resuscitation, his CT of the brain showed the entry site of bullets was the right parietal area, bihemispheric injuries, multilobar injuries, SAH, IVH, midline shift 6 mm to the right. (60 points) as shown in Figure 2. Therefore, he will get a total point value of 131, which approximately corresponds to more than 50-60% probability of death at hospital discharge. 

The calibration plot showed that the model was very close to the ideal Figure 3 and had a bias-corrected c-index value of 0.867. For general application, validation of the predictive nomogram was evaluated as the binary classifiers (death or living at hospital discharge) instead of the predicted probability of death. Therefore, we proposed the optimal cut-off point of the total scores of this nomogram to predict the hospital-discharge mortality. As the results, the scale’s sensitivity, specificity, PPV, NPV were demonstrated in Table 4 and ROCs, and AUCs were revealed in Figure 4. As the results, the optimal cut-off points for predicting hospital-discharge was the 100 scores because this cut-off point which had the highest sensitivity, specificity, PPV, NPV, accuracy and AUC. 

## Discussion

Penetrating brain injury is one of the most challenging clinical entities for neurosurgeons. The present study concerned the mortality of patient with PTBI. Overall mortality rate of the present study was 32.3%, while previous studies reported mortality of PTBI range 21-88%. Causally, our series included various penetrating mechanisms such as a gunshot, secondary blast effect, and a stab wound to the head. Hofbauer *et al*. reported that the mortality rate of a gunshot wound to the head was 87% while the mortality rate of the non-gunshot wound injuries was 4% [7,25]. 

Several studies have been reported the various prognostic factors influencing the mortality of PTBI. Lower GCS, bilateral fixed and dilated pupils are the common the significant prognostic factors which have been reported [26]. Moreover, hypotension and anemia were described that these parameters associated with increased mortality by Decuypere *et al*., [27]. In accordance with the previous studies, the results of the present study revealed the clinical parameters were: lower GCS, bilateral mydriasis, hypotension, and anemia. The absence of pupil reactivity in general conﬁrms the brainstem function cruelly compromised, whereas patients with normal pupil reactivity reflex correlating with an undamaged function of brainstem. Additionally, hemorrhagic shock and anemia can have observed in severe TBI and caused compromised cerebral perfusion [27,28]. Additionally, Maragkos reported suicide-related PTBI is significantly associated with mortality. Conversely, this factor was not significant predictors because of the small proportion of suicidal attempt in our cohort [28]. 

Aspects of the missile/flak track, which have been studied for prognostic value, include the presence of bihemispheric lesions, multilobar involvement, and ventricular involvement. Additionally, cerebral contusion, SAH, IVH, SDH, and evidence of increased intracranial pressure (cistern effacement, and midline shift) related signiﬁcantly with increased mortality and an unfavorable outcome [10,11]. In the same way, the results of the present study were mainly accordant with other studies. 

The predicted model from logistic regression analysis has been presented in various methods, including score chart, web-based calculator, and nomogram. Muehlschlegel *et al*. proposed the SPIN score for predicting survival after PTBI with various options of predicting model [11]. Besides, Bendt *et al*. developed the St. Louis Scale for Pediatric Gunshot Wounds to the Head [10]. The St. Louis Scale were externally validated by Decuypere *et al*., Based on data from the 71 patients in this study, the sensitivity, specificity, positive predictive value, and negative predictive value of the St. Louis scale in predicting death (score ≥ 5) were 94.1%, 75.6%, 78%, and 93.3%, respectively [27]. 

The nomogram is one of methods to present the predicted model as the graphic scoring. We used this method because the nomogram is the simple and easy to implication in the clinical practice. Moreover, we defined the optimal cut-off point of this nomogram for predicting the mortality. As the results, the AUCs showed that 100 scores were the optimal cut-off point for predicting the risk of death. Moreover, the sensitivity, specificity, positive predictive value, and negative predictive value of the present nomogram in predicting mortality (total score ≥ 100) were 80%, 92.6%, 72.7%, 95.0%, and 0.86 respectively. 

Finally, certain limitations of the present study should be acknowledged. The retrospective design may have led to bias and an inability to control confounding factors [29]. However, we tried to tackle this limitation by employing a multivariable analysis. Additionally, the nomogram needs the external validation in the future because this is the first paper proposed the first nomogram of PTBI as our knowledge**. **

In conclusion, PTBI is the fatal injury depend on both clinical and neuroimaging parameters. The nomogram is the alternative method presenting the prognostic model providing prognostic parameters toward implication for clinical decision making.

## Conflict of Interest:

None declared.
